# Antiplatelet Drugs Use in Pregnancy—Review of the Current Practice and Future Implications

**DOI:** 10.3390/jpm14060560

**Published:** 2024-05-24

**Authors:** Nebojsa Antonijevic, Nikola Gosnjic, Marija Marjanovic, Jovana Antonijevic, Milica Culafic, Jovana Starcevic, Milana Plavsic, Danka Mostic Stanisic, Ana Uscumlic, Zaklina Lekovic, Dragan Matic

**Affiliations:** 1 Clinic for Cardiology, University Clinical Center of Serbia, 11 000 Belgrade, Serbia; 2 Faculty of Medicine, University of Belgrade, 11 000 Belgrade, Serbia; 3 Department of Pharmacokinetics and Clinical Pharmacy, Faculty of Pharmacy, University of Belgrade, 11 000 Belgrade, Serbia; 4 Center for Anesthesiology and Resuscitation, University Clinical Center of Serbia, 11 000 Belgrade, Serbia; 5 Clinic for Gynecology and Obstetrics, University Clinical Center of Serbia, 11 000 Belgrade, Serbia

**Keywords:** antiplatelet therapy, pregnancy, risk of bleeding

## Abstract

When clinicians opt for antithrombotic therapy to manage or prevent thrombotic complications during pregnancy, it is imperative to consider the unique physiological state of the pregnant woman’s body, which can influence the pharmacokinetics of the drug, its ability to traverse the placental barrier, and its potential teratogenic effects on the fetus. While the efficacy and safety of aspirin during pregnancy have been relatively well-established through numerous clinical studies, understanding the effects of newer, more potent antiplatelet agents has primarily stemmed from individual clinical case reports necessitating immediate administration of potent antiplatelet therapy during pregnancy. This review consolidates the collective experiences of clinicians confronting novel thrombotic complications during pregnancy, often requiring the use of dual antiplatelet therapy. The utilization of potent antiplatelet therapy carries inherent risks of bleeding, posing threats to both the pregnant woman and the fetus, as well as the potential for teratogenic effects on the fetus. In the absence of official guidelines regarding the use of potent antiplatelet drugs in pregnancy, a plethora of cases have demonstrated the feasibility of preventing recurrent thrombotic complications, mitigating bleeding risks, and successfully managing pregnancies, frequently culminating in cesarean deliveries, through meticulous selection and dosing of antiplatelet medications.

## 1. Introduction

Considering the increasing incidence of pregnancy in older age, the use of antiplatelets by pregnant women makes an important discussion point for many clinicians [1,2]. These drugs are used for many indications, like angina pectoris, myocardial infarction, ischemic cerebrovascular insult, preeclampsia, prevention of placental thrombosis and miscarriage, intrauterine growth restriction (IUGR) associated with placental insufficiency, antiphospholipid syndrome, thrombophilia and secondary prophylaxis after coronary bypass surgery In addition, they are indicated as an additional therapy for the prevention of mechanical valve thrombosis or as a monotherapy in case of biological heart valves three months after surgery (aspirin only), and in sticky platelets syndrome [1,3,4,5].

Besides understanding the pathology for treating any of these diseases, clinicians should always be aware of the common pharmacokinetic changes affecting this special population of patients. A rise in the activity of liver enzymes, an increased degree of glomerular filtration and plasma volume, changes in protein binding and reduction in serum albumin levels should always be accounted for in pregnant women [1]. In addition, various risk factors such as smoking, hypertension, diabetes, dyslipidemia, acquired thrombophilic conditions (antiphospholipid antibody syndrome and hyperhomocysteinemia), thrombophilia (acquired or inherited), infections, etc., that may cause cardiovascular pathologies in pregnant women should also be assessed on an individual patient basis [1].

In the following paper, we first give an overview of the general pharmacokinetic and pharmacodynamic changes occurring in pregnancy and then we present antiplatelet drugs, focusing on their efficacy and safety when used in pregnancy and lactation.

### Literature Search and Study Selection

The methodology employed in this review paper involved a structured approach to identify and incorporate the pertinent literature. Initially, a well-defined research question was formulated to delineate the scope of the review. Subsequently, a comprehensive search strategy utilizing relevant keywords was developed for Scopus database searches. Search results were screened based on title and abstract relevance, followed by a thorough assessment of full-text articles to ensure their suitability for inclusion. Pertinent data were extracted from selected articles, and their quality was evaluated using established criteria (study design appropriateness, methodological rigor, control of bias and confounding, validity and reliability of measurements, adherence to ethical considerations, transparency in statistical analysis, and generalizability of findings). Synthesis and analysis of findings from the included studies were conducted to address the research question.

## 2. Pharmacokinetic Changes in Pregnancy

Given the fact that most clinical studies exclude pregnant women, caution should always be practiced when deciding on the use of drugs in pregnancy. Understanding pharmacokinetic changes can be a significant piece of a challenging puzzle to be accounted for in this complex patient population.

### 2.1. Drug Absorption

Although clinical significance of the changes occurring in drug absorption in pregnancy are not clear, it is well known that gastric acid production is decreased in pregnant women. The impact of this change will depend on the drug properties—i.e., weak acids such as aspirin might have reduced absorption due to increase in their ionization [6]. Due to increase in plasma progesterone values, gastrointestinal motility is reduced by up to 50%, which can be clinically relevant when rapid onset of drug action is needed [7]. On the other hand, due to increase in cardiac output and consequent increase in intestinal blood flow, these effects are countered, leading to relatively minimal changes in the overall absorption. However, nausea and vomiting occurring typically in the beginning stages of pregnancy will unequivocally lead to a decrease in drug absorption. Patients experiencing nausea and vomiting should be advised to take their drugs at times of day when nausea is minimal—i.e., for most patients, in the evening [6,7].

### 2.2. Drug Distribution

Cardiovascular changes such as increased cardiac output and stroke volume might affect drug distribution. In addition, pregnancy results in an increase by up to 42% in the plasma volume followed by an increase in overall body water in all compartments. By the late third trimester, the plasma volume increases by more than 50–60% [6]. The total increase in body water is approximately 8 L, out of which 60% is distributed to placenta, fetus, and amniotic fluid and the remaining 40% to maternal tissues [7]. This might cause increased distribution and lower plasma concentration for hydrophilic drugs (i.e., drugs with a relatively low volume of distribution). Moreover, distribution of lipophilicity might also be considered elevated since maternal body fat increases by around 4 kg. On the other hand, plasma protein binding decreases, leading to drugs being more freely distributed in the tissues. Blood flow to the uterus should also be taken into consideration since fetus and amniotic fluid act as an additional compartment.

### 2.3. Drug Metabolism

During pregnancy, significant alterations occur in drug metabolism, primarily driven by changes in the activity of various drug-metabolizing enzymes. Notably, the activity of certain cytochrome P450 (CYP) enzymes, including CYP3A4, CYP2A6, CYP2D6, and CYP2C9, is increased, while other enzymes such as CYP1A2 and CYP2C19 exhibit decreased activity. The impact of these changes on drug metabolism varies depending on the form of the administered drug, whether it is directly active or a pro-drug requiring metabolic activation. Additionally, phase II enzyme activity is also affected during pregnancy, with a substantial increase observed in UDP-glucuronosyltransferase 1A4 (UGT1A4) activity. Specifically, UGT1A4 activity increases by 200% during the first and second trimesters and by 300% during the third trimester, leading to accelerated metabolism and decreased concentrations of drugs that are substrates for this enzyme. These dynamic alterations in drug-metabolizing enzyme activity highlight the need for careful consideration of pharmacokinetic changes during pregnancy to ensure optimal drug dosing and efficacy while minimizing potential adverse effects [6].

### 2.4. Drug Excretion

During pregnancy, renal physiology undergoes significant adaptations to support the growing fetus. While glomerular filtration increases by approximately 50% in the first trimester and continues to rise until the final weeks before delivery, alterations in tubular transporters play a crucial role in determining drug excretion rates. These changes in tubular transporters can result in variable effects on drug excretion, leading to complex pharmacokinetic profiles for many medications. Some drugs may exhibit increased renal clearance due to enhanced tubular secretion, while others may show reduced clearance as a result of altered reabsorption or secretion processes. Furthermore, the expression and activity of specific transporters may vary among individuals and throughout different stages of pregnancy, further complicating the prediction of drug excretion patterns. Therefore, while glomerular filtration rate changes serve as an important indicator of renal function during pregnancy, the overall impact on drug excretion cannot be generalized, highlighting the need for individualized pharmacokinetic considerations in pregnant patients [6].

### 2.5. Drug Transport through Placenta

Typically, xenobiotics cross the placenta by simple diffusion. Many molecular characteristics such as protein binding, solubility in lipids, ionization degree and molecular weight play an important role in crossing natural barriers including the placenta by simple diffusion. In general, molecules that are small, lipid-soluble, and unbound can cross the placenta. Other molecules can cross it only via active pathways (i.e., via transporters) if they share molecular resemblance to endogenous molecules transported in this way such as cytokines or steroid hormones [6].

## 3. Antiplatelet Drugs in Pregnancy

### 3.1. Brief Pharmacological Classification, Indications, and Considerations

In pregnancy, various pathological conditions necessitate the use of antiplatelet drugs, which inhibit platelet aggregation so as to mitigate thrombotic risks. These medications are categorized under the Anatomical Therapeutic Chemical (ATC) classification system as B01AC, Platelet aggregation inhibitors excluding heparin. Aspirin (acetylsalicylic acid), classified as B01AC06, is useful for preventing preeclampsia in high-risk women, managing antiphospholipid syndrome, preventing recurrent pregnancy loss, and addressing specific cardiovascular events. Clopidogrel (B01AC04) may be cautiously considered in select cases, such as preventing stent thrombosis in pregnant women with coronary artery stents, although data supporting its use in pregnancy are limited. Ticagrelor (B01AC24) and Prasugrel (B01AC22) are generally avoided during pregnancy due to insufficient safety data. The decision to employ antiplatelet therapy in pregnancy necessitates individualized risk assessment, close monitoring, and collaboration among healthcare professionals to optimize maternal and fetal outcomes.

### 3.2. Aspirin

Aspirin has a long history of clinical use in various patient populations, including pregnant women. We present below some of the studies we considered most relevant for aspirin use. Important to note for aspirin is its ability to cross the placenta, so the safety from the perspective of both mother and fetus should be considered.

As shown in the large meta-analysis that included 32,217 pregnant women, the use of 50–150 mg of aspirin as a monotherapy (as used by 98% of the women whereas the rest used combined therapy with dipyridamole) does not pose a statistically important risk of developing postpartum hemorrhage in mothers. Postpartum hemorrhage that was defined as bleeding equal or greater than 500 mL, occurred in 15% of patients, regardless of whether they used aspirin or not [8]. Another significant review article showed that the use of either dipyridamole or low-dose aspirin does not carry a statistically important risk of causing placenta abruption when compared to placebo [9].

More than twenty years ago, the results of a big, randomized, double-blinded study in which aspirin (60 mg) was used as a treatment for preeclampsia showed it to be safe from the aspect of congenital malformations, major motor deficit, and severe neuromotor or developmental delay both during pregnancy and in early childhood [10]. However, although used in the aforementioned study, aspirin in as low a dose as 60 mg, is not readily available. Typically, small dosages that are available are those of 75 mg, 81 mg and 100 mg.

A significant concern for many clinicians choosing to prescribe aspirin for pregnant women is its effect on the closure of the ductus arteriosus. In an in situ study of 511 stillborn where mothers used aspirin antenatally, 15 were found to have had premature closure of the ductus arteriosus. However, the conclusion of the study was that premature closure of the ductus arteriosus occurred because of a combination of various factors—intrauterine infection (60%), umbilical cord abnormalities (67%), and retroplacental hemorrhage (87%)—but was not caused by aspirin [11]. In other studies, Doppler blood flow measurement was used to assess the blood flow through the fetal ductus arteriosus. One such study compared placenta blood flow in 106 pregnant women who did not take aspirin with 65 pregnant women who were under treatment with low doses of aspirin (80 mg or 100 mg). The results showed that low-dose aspirin intake did not influence hemodynamics in the fetal ductus arteriosus in these two groups [12].

One of the most common indications for the use of aspirin in pregnancy is preeclampsia in which aspirin proves to be not only relatively safe, but also and especially to be effective in achieving better pregnancy outcomes. A recent meta study included the results of 3168 pregnancies from randomized clinical trials in which aspirin was used in the early stages of pregnancy (week 16 or earlier) that carried the risk of preeclampsia. Results of this analysis showed that when aspirin was used, positive pregnancy outcomes were achieved both for mother (gestational hypertension and premature delivery) and for the newborn (intrauterine growth retardation, intrauterine death and decreased baby weight and development). A subgroup of 864 babies had 110.44 g higher body weight when aspirin was used as compared to placebo [13].

On the other hand, a meta-analysis that included eight studies showed that the use of aspirin during the first pregnancy trimester may cause gastroschisis more often in the exposed babies. However, the adequacy of studies under consideration was deemed debatable. In one of these studies, the mothers were of low socioeconomic status and abused certain drugs. Other antipyretics may have caused the gastroschisis in another of the studies; and in two more of the analyzed studies, the problem of objectivity was stressed since healthy babies were used as the control group [14].

It is important to note that some of the literature articles mention that the use of aspirin during pregnancy may lead to orofacial cleft (cleft lip and cleft palate). Although such a risk cannot be completely disregarded, the study examining reports from the Hungarian national registry of congenital anomalies in 3415 babies showed that orofacial cleft is not more common in newborns whose mothers used aspirin during pregnancy. The same study showed similar results for neural tube defects and gastroschisis [15].

Based on the available safety and efficacy data, the European Society of Cardiology (ESC) Scientific Document Group developed clinical guidelines regarding the use of aspirin in pregnancy and accepted the use of low dose aspirin when indicated in pregnant women. The use of aspirin in pregnancy is presented in Table 1 (adopted from the 2018 ESC Guidelines for the management of cardiovascular diseases during pregnancy) [1,16].

Besides previously mentioned European guidelines, the American College of Chest Physicians (ACCP) also provides a set of recommendations for the use of aspirin during pregnancy which can be seen in Table 2 [16,17].

Another significant indication for the scope of this article is the use of assisted reproductive technology (ART). For this indication, low doses of aspirin are used for varying duration. However, despite its wide use in this therapy, evidence supporting its effectiveness for the primary end outcome of live births was not shown in the recent systematic review which collected data from thirteen randomized clinical trials involving a total of 2653 participants [18].

One possible adverse effect of aspirin that is always significant regardless of the treated population is upper gastrointestinal toxicity. We did not identify studies specifically focusing on pregnant women experiencing upper gastrointestinal bleeding due to aspirin, but data from the general population is available. A study which included 10,000 subjects showed that upper gastrointestinal symptoms occurred in 15.4% of patients receiving low dose aspirin (doses ranging from 75 to 325 mg), with the most common complaint being gastroesophageal reflux (approximately 70% of reports) [19]. However, clinical judgement should be used when assessing this kind of data. Prior history of dyspeptic disorders was still the most important predictor of possible gastrointestinal adverse effects. Also, discontinuing low dose aspirin in patients with a significant cardiovascular disease risk might cause life threatening thromboses and increase their overall mortality [20]. In these cases, clinicians often use prophylactic treatment for the gastrointestinal symptoms caused by aspirin, with proton pump inhibitors (PPIs) being the most common option. However, these drugs also might cause detrimental effects on pregnancy as shown by recent meta-analysis indicating that PPI may carry an increased risk of causing congenital malformations of the fetus. However, such malformations were not specified and it was shown that their use does not correlate with miscarriage, neonatal death and preterm delivery [21]. When taken in the early stages of pregnancy, PPIs are considered to possibly cause either early or late preeclampsia. Interestingly, if used after the 28th week of gestation, these drugs caused a decrease in the risk of developing preterm (earlier than 37th pregnancy week) or early (earlier than 34th pregnancy week) preeclampsia, which may be a consideration for their future use [22].

As a conclusion, although aspirin has a long history of use in pregnancy and is considered generally safe, considering the fact that it crosses the placenta [23] and all other specificities of the given population and the drug itself, its use requires a careful approach.

### 3.3. P2Y12 Receptor Antagonists

The first line of treatment in patients experiencing signs of ischemia and coronary artery disease as well as in those in whom a large viable myocardial territory is at risk (e.g., proximal left anterior descending (LAD) coronary artery disease) is the percutaneous coronary intervention (PCI) [24]. For this reason, understanding the use of the most prevalent pharmacotherapy accompanying PCI—i.e., dual antiplatelet therapy consisting of aspirin and another antiplatelet drug (most often P2Y12 receptor antagonists) in this high-risk patient population is of utmost importance [25]. These therapies should be understood both from their efficacy and safety aspects, as the combined antiplatelet treatment might pose an additional bleeding risk for patients. Although not often used in daily clinical practice, a platelet function test may help guide the safe use of antiplatelet drugs and it is presented later in the text below, after the description of the remaining drug classes [26,27].

#### 3.3.1. Clopidogrel

Considering well established clinical practice and uneventful case reports available in the literature, clopidogrel use is considered adequate in pregnancy when dual antiplatelet treatment is indicated.

According to the Summary of Product Characteristics (SmPC) for clopidogrel, neither direct or indirect detrimental effects on the pregnancy course, embryonal, fetal or postnatal development were shown in the animal studies. Although the SmPC states that clopidogrel is not recommended in pregnant women due to lack of randomized trials, based on the current publications, clopidogrel is considered to be the safest drug from the group of adenosine diphosphate (ADP) receptor inhibitors [28]. In the following section, we give a short description of several clinical case reports.

The first case report presented here describes a thirty-nine-year-old pregnant woman experiencing myocardial infarction in the sixth week of gestation, who was treated with percutaneous coronary intervention including the use of aspirin and clopidogrel until delivery. The baby was born with an open foramen ovale (0.2 mm), restrictive communication of interventricular muscles and moderate mitral insufficiency. However, such complications were not deemed to be related to the clopidogrel use [29].

Moreover, other available cases from the literature attest to the safe use of clopidogrel in pregnancy. One older case report from 2008 shows a 44-year-old pregnant woman experiencing acute myocardial infarction (AMI) at week 8 of gestation. She was treated with PCI using a drug-eluting stent in combination with eptifibatide (bolus of 180 μg/kg, followed by a 2 μg/kg/minute intravenous infusion for the following 24 h), aspirin and clopidogrel (loading dose of 600 mg). The patient was discharged on 75 mg of clopidogrel and 100 mg of aspirin daily. No complications were observed and a healthy infant was delivered at 36th week of gestation after a cesarean section, having withheld aspirin and clopidogrel on the morning of surgery [30]. Another case report with similarly positive results from 2009 describes a 43-year-old woman experiencing AMI at the 21st week of gestation. She was treated with the implantation of a bare metal stent followed by the initiation of aspirin (100 mg daily) and clopidogrel (75 mg daily for two weeks). An elective cesarean section was performed at week 32 of gestation, with the treating clinicians deciding to discontinue aspirin 7 days prior to the surgery and replace it with heparin, despite the fact that heparin is not an adequate substitution for aspirin since it belongs to a different group of medication. Nonetheless, the delivery was uneventful and a healthy baby was born [31].

Reilly et al. presented a case of the use of clopidogrel in a 33-years-old woman with a history of multiple prior ischemic strokes and transient ischemic attacks. The patient was started on clopidogrel as a secondary prevention for stroke prophylaxis prior to conception (having been on clopidogrel treatment for 7 years prior to conception) and presented to clinic at week 39 with contractions. At the presentation, clopidogrel was discontinued and an induced vaginal delivery was performed 7 days later as the patient refused a cesarian section. A healthy baby was delivered without any delivery complications [32].

According to current recommendations of the ESC, the use of clopidogrel is allowed in pregnancy as a treatment of AMI for the shortest possible period and in cases where other treatments cannot be used [1]. A similar opinion is given by the American College of Cardiology, which states that clopidogrel can be used for the shortest possible duration, with the obligatory discontinuation seven days prior to neuraxial anesthesia in order to prevent epidural hematoma [33].

However, although relatively safe considering the long-standing clinical experience of its use, some of the common problems encountered with clopidogrel such as response variability and resistance to its use prompted clinicians to prescribe more potent P2Y12 inhibitors such as ticagrelor and prasugrel [34] which are described in the following sections.

#### 3.3.2. Ticagrelor

Since ticagrelor and prasugrel represent P2Y12 drugs of choice in combination with aspirin in patients initially treated with PCI (either stent or balloon angioplasty) [35], cases showing their successful use in pregnancy are described next, despite the fact that ticagrelor is not recommended during pregnancy according to the SmPC [36].

In the course of performing the literature search, we identified four cases describing the use of ticagrelor during pregnancy. The first case presents a female patient with a history of Behcet disease experiencing non-ST elevation myocardial infarction (NSTEMI) which was treated with implantation of a drug-eluting stent through percutaneous coronary intervention with dual antiplatelet therapy (DAPT) consisting of aspirin and ticagrelor. These drugs were used for a period of one year—aspirin at the dose of 80 mg and ticagrelor at the dose of 180 mg (2 × 90 mg). Although conception prevention was advised to the patient, she became pregnant 4 months after NSTEMI and decided to continue with the pregnancy. During the pregnancy, she was treated with prednisolone (8 mg), aspirin (80 mg) and bisoprolol (5 mg), all administered once daily. Besides these drugs, the patient also received ticagrelor at the dose of 180 mg (2 × 90 mg) daily, with the plan of continuing it for eight months and discontinuation seven days prior to planned delivery. The patient was regularly examined and abnormal findings were not observed. Ticagrelor therapy was continued as per the described plan, being discontinued two weeks prior to delivery. The delivery was inducted and a healthy baby was born. Histology testing of the placenta was performed without finding any lesions. However, iron loaded macrophages in the decidua were observed, indicating antenatal hemorrhage which was not clinically detectable [37].

The next identified case shows a 34-year-old woman presenting with a complete superficial and deep venous intracranial sinus occlusion in her 10th week of pregnancy. Despite being treated with anticoagulants (enoxaparin 80 mg s.c. b.i.d.), the patient’s state deteriorated and she became comatose. After unsuccessful rheolysis and balloon angioplasty, monitored heparinization was continued and aspirin (500 mg, i.v.) was introduced two days later. As the state of the patient remained unchanged, she was started on eptifibatide on day 14 of admission. The patient regained consciousness after six days, and eptifibatide was continued for the following two weeks (according to the SmPC, the drug should be used for up to 96 h), after which two doses of 180 mg ticagrelor daily were initiated, whereas aspirin was continued without making any dose adjustments. She was discharged 26 days after endovascular treatment with undisturbed pregnancy. The aspirin dose was reduced at week 30 of her pregnancy to 300 mg daily per os. Seven days prior to delivery, ticagrelor was stopped, whereas aspirin and eptifibatide were discontinued two days prior to delivery. An uncomplicated cesarian section was performed at week 38 of gestation with the delivery of a healthy baby. Aspirin and anticoagulant therapy were later reintroduced. Important to note about this case is the fact that treating clinicians performed monitoring of antiplatelet therapy (i.e., aspirin and ticagrelor using Multiplate (Roche Diagnostics, Munich, Germany), VerifyNow (Accriva, San Diego, CA, USA), and the PFA-200 system (Siemens, Erlangen, Germany) platelet function tests [38].

Another report on the use of ticagrelor during pregnancy describes a 37-year-old woman developing AMI in the 27th week of gestation. The patient was treated with intravenous aspirin (500 mg), enoxaparin (10,000 IU), a loading dose of ticagrelor (180 mg) and subsequently had two drug-eluting (sirolimus) stents implanted on the left main–left anterior descending coronary artery and additional paclitaxel-eluting balloon expansion on the ostial-proximal portion of the first diagonal branch. Due to the high thrombotic burden, unfractionated heparin and eptifibatide were introduced until achieving TIMI (Thrombolysis in Myocardial Infarction) 3 flow. The patient was discharged with dual antiplatelet therapy consisting of aspirin and ticagrelor. After twelve weeks, the patient was admitted to hospital in order to have the cesarean section performed. In accordance with the recommendations on perioperative management of P2Y12 antagonists [34], dual antiplatelet therapy with ticagrelor was discontinued five days prior to delivery and bridging therapy with tirofiban was administered (intravenous tirofiban at the dose of 0.4 mg/kg/min during 30 min, followed by 0.1 mg/kg/min for the next 96 h until 4 h prior to delivery). A cesarean section was performed and a healthy baby was born without any complications. A subtotal hysterectomy for the atonic postpartum hemorrhage was also performed. Tirofiban was continued two hours after the surgery and stopped after ten hours, at which time dual antiplatelet therapy with the loading dose of 180 mg of ticagrelor was reintroduced. The patient was advised to continue dual antiplatelet therapy for the period of one year. On the follow up that occurred 27 weeks after the delivery, both the patient and the baby were in good condition [39].

With the shown cases, we would like to stress that up to the date of this publication, all clinicians opted for the discontinuation of ticagrelor and for bridging therapy with GP IIb/IIIa antagonists, in accordance with the recommendations of the ESC. However, an appropriate team of clinicians experienced in the use of antiplatelet therapy may opt for a different approach based on the type of surgery and the current clinical course of the individual patient.

Recently, we presented a case of a 32-years old pregnant women experiencing large AMI of the anterolateral wall, complicated by cardiogenic shock in the 38th week of pregnancy. The patient was treated with a drug-eluting stent implanted in the osteo-proximal part of left anterior descending coronary artery and dual antiplatelet therapy consisting of 300 mg of aspirin and 180 mg of ticagrelor. Less than 24 h after a myocardial infarction, delivery started, and an urgent cesarean section was indicated. As multiplate aggregometry testing showed a relatively insufficient level of ticagrelor platelet inhibition and a moderate level of aspirin platelet inhibition, a cesarean section was performed without discontinuation of ticagrelor, due to the need for emergency surgery. Local hemostatic measures including administration of tranexamic acid were applied. The procedure passed without complications and a healthy male baby was born. Taking into consideration mild epistaxis and blood in lochia secretion in the subsequent treatment course, it was decided to replace ticagrelor with clopidogrel. However, after receiving a loading dose of 600 mg of clopidogrel in two divided doses, aggregometry testing showed even more significantly pronounced clopidogrel resistance. Therefore, clopidogrel was discontinued and ticagrelor was reintroduced. In the following course of the following days, aggregometry testing showed adequate response to ticagrelor. To the best of our knowledge, this is the first reported case of surgery in pregnant women treated with dual antiplatelet therapy without ticagrelor discontinuation [40].

Since current clinical experience of using ticagrelor in pregnancy is scarce, albeit positive, the ESC advises against its use in this patient population until more information is available [1].

#### 3.3.3. Prasugrel

According to the SmPC for prasugrel, its use in pregnancy is not recommended unless other treatment cannot be used. Although animal studies have not shown negative effects on embryonal, fetal or postnatal development, clinical studies are still not available [41]. However, although its routine use is not recommended in pregnancy if there are alternative treatment options, case reports of its successful use in pregnancy do nevertheless exist. During our search, we identified two such cases.

In a report from 2015, prasugrel was used as part of dual antiplatelet therapy in combination with aspirin after implantation of two drug-eluting stents in a 32-year-old woman experiencing NSTEMI in the early stage of pregnancy. Prasugrel was initially administered at a loading dose of 60 mg and was subsequently used at the maintenance dose of 10 mg daily, whereas aspirin was given at the dose of 81 mg daily. Pregnancy was discovered one month after DAPT initiation. The patient decided to continue with pregnancy concomitantly using dual antiplatelet therapy. Follow-up examinations were without any abnormal findings, so planned induction and delivery were scheduled accordingly. DAPT was discontinued five days prior to delivery. We point out that the ESC recommends discontinuing prasugrel seven days prior to surgery [35]. A cesarian section was performed at 38 weeks and 5 days of pregnancy without any complications. A normal-phenotype baby was born without hemorrhage or signs of any malformations whatsoever. The mother also had not experienced any complications, so the dual antiplatelet therapy was re-introduced in the aforementioned maintenance regimen [42].

Another, more recent case shows successful delivery in a patient treated with prasugrel during pregnancy. The case describes a 41-year-old woman diagnosed with unruptured carotid-ophthalmic aneurysm in the 12th week of pregnancy. Clinicians decided to perform stent-assisted embolization of the aneurysm. As the preoperative testing showed clopidogrel resistance, the patient was pre-medicated with aspirin (75 mg) and prasugrel (“off-label” regimen 2 × 10 mg) one day prior to the surgery. The embolization was performed in the 17th week of pregnancy, without any peri-operative complications. For the following five months, the aforementioned antiplatelet therapy was continued. Prasugrel was stopped two weeks prior to full term whereas aspirin was continued. Labor was induced at week 39 of gestation and an uneventful vaginal delivery ensued. The routine follow-up 3 months after birth did not show any abnormalities [43].

Despite the positive experience described above, the ESC still does not recommend the use of prasugrel in pregnancy until further evidence supporting its safe use emerges [1].

#### 3.3.4. Ticlopidine

Ticlopidine was one of the first used P2Y12 antagonists but it is now rarely used due to rare but serious adverse effects such as neutropenia/agranulocytosis, thrombotic thrombocytopenic purpura and aplastic anemia [44]. A further reason favoring the use of the newer P2Y12 antagonists is the slower onset of action of ticlopidine [45].

In the preparation of this review, only one article describing the use of ticlopidine in pregnancy was identified. This case presents a woman with mechanic aortic valve on warfarin therapy presenting with the intention of becoming pregnant. Warfarin was discontinued due to the planned pregnancy and replaced with dipyridamole (300 mg daily), ticlopidine (300 mg daily) and aspirin (81 mg daily). In the 36th week of pregnancy, the patient was admitted and these drugs were discontinued and replaced with heparin. A cesarean section without complications was performed at week 38 of gestation with the delivery of a healthy baby [46].

However, safer alternatives with more rapid onset of action are available, making ticlopidine a rarely used drug in modern clinical practice.

#### 3.3.5. Cangrelor

Cangrelor stands as the singular intravenous platelet P2Y12 receptor inhibitor currently available. It distinguishes itself through its potent, predictable, and reversible antiplatelet effects. Extensively evaluated within the CHAMPION (Cangrelor Versus Standard Therapy to Achieve Optimal Management of Platelet Inhibition) program, cangrelor underwent comparison with various clopidogrel regimens, establishing its indication for employment in patients with coronary artery disease undergoing percutaneous coronary intervention [47]. Cangrelor, characterized by a brief half-life of approximately six minutes, facilitates swift reinstatement of platelet functionality. Its application as a bridge therapy preceding coronary artery bypass graft surgery in patients with cardiac stents has been delineated across various surgical disciplines [48]. Pertaining to pregnancy considerations, the existing clinical data concerning safety of cangrelor’s use during pregnancy remains limited. In the context of cesarean delivery, which entails a potential risk of substantial blood loss, cangrelor emerges as a notable agent owing to its prompt effect.

A recent publication marks the inaugural documentation of cangrelor’s administration in a pregnant individual. In this case, a 34-year-old woman at 31 weeks’ gestation necessitated intracranial stenting due to an acute ischemic stroke. As part of her anticoagulant regimen to forestall stent occlusion, she initially received aspirin and clopidogrel. Subsequently, five days preceding the cesarean section, clopidogrel was substituted with cangrelor, which was discontinued upon induction of anesthesia. The surgical procedure and subsequent recovery phase transpired without complications. Cangrelor served as a bridging therapy during the transition from clopidogrel, thereby minimizing blood loss in the patient [49].

However, in light of the dearth of established safety data concerning the effects of cangrelor on maternal and fetal well-being, its utilization during pregnancy is cautioned against, unless the projected advantages substantially surpass the potential hazards [50,51].

### 3.4. GP IIb/IIIa Inhibitors (Tirofiban, Eptifibatide and Abciximab)

This class of antiplatelets has not been widely used in pregnancy and should therefore not be used unless absolutely needed, with the most common indications being periprocedural use in patients with prior myocardial infarction, high thrombus burden and complex PCI [52]. When used during pregnancy, the preferred method of delivery should be cesarean section in order to avoid potential fetal intracranial hemorrhage that might occur in vaginal delivery [53].

#### 3.4.1. Tirofiban

According to the SmPC for tirofiban, its use is not recommended in pregnancy unless clearly necessary [54]. Data regarding the use of tirofiban in pregnant women is mostly insufficient and relies on a number of anecdotal reports. The most recent case of its use in pregnancy is described above in the ticagrelor section, when bridging tirofiban therapy was administered and the delivery was performed without any negative effects to the newborn or the mother [39].

Oral antiplatelet therapy bridging with tirofiban was shown to be both a safe and effective therapeutic option for patients requiring surgery during oral antiplatelet use [55].

Only two additional cases describing the use of tirofiban in pregnant women are presented in the literature. One of these two articles depicts a woman at the 25th week of pregnancy being hospitalized due to an increased risk of premature labor. As the patient had a history of pulmonary embolism, she was started on enoxaparin. However, on day 7 and 9, she suffered from heparin-induced thrombocytopenia and pulmonary embolism, respectively. An intravenous bolus of tirofiban was used in combination with heparin as a treatment of the pulmonary embolism, together with a pulmonary embolectomy under cardiopulmonary bypass (CPB). After instituting CPB, tirofiban was used as a continuous infusion at a dosage of 0.15 mg/kg/min and the thrombus was extracted. The postoperative course was uneventful both for mother and fetus. Tirofiban was discontinued and anticoagulation was managed with fondaparinux. Elective delivery of the healthy baby was performed at week 38 [56].

The third available case shows a pregnant woman experiencing an acute myocardial infarction in the 20th week of gestation. Tirofiban was used combined with heparin due to significant thrombus load, and TIMI 3 flow in LAD was achieved. Then, 24 h after tirofiban infusion, catheterization (kissing balloon inflations in LAD) was performed. No complications were observed for mother and fetus in the period of 5 days after the procedure, so the patient was discharged, with aspirin and clopidogrel used as DAPT prophylaxis [57].

According to previously mentioned case reports, tirofiban has not caused any adverse event to pregnant women, their fetus or the newborn. However, all authors accentuate the necessity of the inclusion of a multidisciplinary team in the final therapy decision. In addition, we highlight the importance of limiting the duration of tirofiban administration due to the risk of developing thrombocytopenia [54].

#### 3.4.2. Eptifibatide

According to the SmPC, eptifibatide should not be used in pregnant women unless clearly needed as the clinical data elucidating its use in pregnancy is scarce [58].

According to our literature search, three cases of use of eptifibatide in pregnancy are available. The most recent one was presented in connection with ticagrelor administration, demonstrating successful simultaneous use of eptifibatide [38], and here we present the other two cases where eptifibatide was used after pregnant women experienced acute myocardial infarction.

In the first case, the patient’s treatment consisted of a bare metal stent insertion combined with aspirin and clopidogrel medication. Clopidogrel was discontinued 4 weeks prior to delivery. As the patient had high risk of stent thrombosis, eptifibatide was used as a bridging therapy for 7 days and was stopped for the labor induction, although as per the eptifibatide SmPC, the drug should only be used up to 96 h. Operative vaginal delivery was performed without any complications [59].

The second case refers to a pregnant woman in her 8th week of gestation experiencing an acute myocardial infarction treated with a drug-eluting stent and use of eptifibatide bolus (180 μg/kg), followed by a 2 μg/kg/minute intravenous infusion for the next 24 h. After continuing subsequent treatment with aspirin and clopidogrel, a cesarean section was performed in the 36th week of gestation, without any complications [30].

#### 3.4.3. Abciximab

Abciximab should be used in pregnant women only if clearly needed as it is not evident whether it has detrimental effects on fetuses or pregnant women as neither studies in humans nor in animals are available. Its effects are short, with the platelets recovering their full function after administration of the 48 h course of treatment [60]. The ESC does not recommend the use of abciximab in pregnant women as it is not known whether the drug crosses the placenta or if it is excreted in milk [1].

### 3.5. PDE3 Inhibitors

#### 3.5.1. Cilostazol

Cilostazol is a drug indicated in the treatment of intermittent claudication [61] and is also, albeit very rarely, used “off-label” as part of triple antiplatelet therapy together with aspirin and P2Y12 antagonists [62,63].

According to the SmPC, cilostazol must not be used in pregnancy. Studies in animals have shown its reproductive toxicity. It is not known whether the drug crosses the placenta or if it is secreted in milk [61].

According to the literature search, only one case report of the use of cilostazol was identified. The case presents a woman in the 24th week of pregnancy experiencing incessant polymorphic ventricular tachycardia (PVT) failing to respond to quinidine, magnesium, isoproterenol, amiodarone, esmolol, and cilostazol during her PVT storm [64]. Of note, cilostazol was used due to its anti-ventricular-fibrillation potential in Brugada syndrome [65]. On hospital day 3, an emergency cesarean section was performed and a limp and apneic male infant was delivered and urgently transferred to the Neonatal Intensive Care Unit. The mother was stabilized with the use of verapamil and amiodarone and was discharged on hospital day 19, whereas the infant was discharged at a full-term corrected gestational age. Unfortunately, the baby passed away at his 5th month of age. Cause of death was deemed as secondary to sudden cardiac death [64].

#### 3.5.2. Dipyridamole

Dipyridamole is not prohibited in pregnancy since it has been used for years without ill consequence, according to the SmPC. Also, as per the SmPC, animal studies have not shown detrimental effects [66].

Recent meta-analysis included 27,510 women randomized either to low dose aspirin–dipyridamole or placebo–no treatment as prevention of preterm birth. Dipyridamole proved to be effective in reducing the risk of premature birth, either when used as monotherapy or in combination with aspirin [67].

Similar results were noted in a review study, describing dipyridamole use in combination with heparin, in terms of reducing perinatal mortality, preterm birth before 34th and 37th week of gestation, and reduced infant birthweight [68]. Neither one of these studies mentioned significant safety events occurring to mother or the newborn.

It is worth noting that the standard-release form of dipyridamole has pH-dependent absorption that may be decreased when combined with gastric acid suppressors such as PPIs. However, modified release forms of dipyridamole are not affected by this interaction [69].

## 4. Summary of the Use of Antiplatelet Therapy in Pregnancy

The most important characteristics of use of antiplatelet therapy in pregnancy are presented in Table 3 which is adapted according to the ESC Guidelines for the management of cardiovascular diseases during pregnancy [1]. Aspirin is a widely used antiplatelet drug during pregnancy, with no effect on the fetus at dosages of 100 mg or less and a small secretion in milk. Clopidogrel could be used if there is need for P2Y12 blockers but implies avoiding breast feeding. There is still not enough existing data about the use of ticagrelor or prasugrel in pregnancy, although the latest results support the usage of these drugs in late periods of pregnancy.

Tirofiban and eptifibatide could be used in the late stage of pregnancy if it is necessary, but with caution. Abciximab is not recommended in pregnancy due to its ability to cross the placenta and its excretion in milk. There is still no data about treatment with abciximab. Cilostazol is not recommended for use in pregnancy due to its reproductive toxicity observed in animal studies, while dipyridamole can safely be used.

## 5. Future Implications

Acute thrombotic events and pregnancy are not rarely associated, especially since pregnancy is now more common later in life. The relationship between antithrombotic therapy, bleeding and recurrent thrombotic events is extremely complex. It is necessary to assess carefully the risk of thrombosis versus the risk of bleeding according to the individual characteristics of each single patient. These risks are of great importance, especially when emergency surgical intervention needs to be carried out immediately after primary PCI, such as an urgent cesarean section in women on dual antiplatelet therapy. Continued efforts should be made to find new therapeutic approaches notably in primary PCIs in pregnancy, that would minimize the risk of bleeding without increasing the risk of thrombotic complications.

## 6. Conclusions

Use of antiplatelet therapy in pregnancy requires well-informed clinical judgement. When considering the above medication, clinicians should always evaluate the dosage regimen and the duration of therapy based on the summary of product characteristics, appropriate guidelines, and current clinical experiences. Furthermore, a thorough understanding of the risk for the mother and fetus on the one side and the potential benefit on the other may guide their adequate use. According to our study, aspirin remains the mainstay drug among antiplatelets due to long clinical experience. Clopidogrel can be considered as an alternative when indicated, as the probably safest option in the class of P2Y12 antagonists. However, as presented in this review, in certain delicate cases, clinicians may opt to use other P2Y12 antagonists. Indications for their use in pregnancy are typically complex and should be managed by the interdisciplinary clinical team. As the evidence regarding more potent antiplatelet drugs emerge, current practice may follow the fast developments in the field and evolve to meet the need for the use of more potent antiplatelets.

## Figures and Tables

**Table 1:** ESC Guidelines for the use of aspirin in pregnancy.

Guideline/Comment	Indication	Class of Recommendation	Level of Evidence
Low dose acetylsalicylic acid (100–150 mg daily) has been recommended for pregnant women with a moderate to high risk of developing preeclampsia from 12th to 36th or 37th weeks.	Hypertension and pre-eclampsia	I	A
In patients with coronary artery disease treated with stent implantation, acetylsalicylic acid in combination with clopidogrel represents the antiplatelet therapy of choice.	Coronary artery disease	/	/
The addition of low dose acetylsalicylic acid to vitamin K antagonists or heparins in pregnant women with prosthetic valves has no proven advantage in preventing valvular thrombosis, but may contribute to the risk of maternal bleeding, including fatal side effects.	Prophylaxis in mechanical prostheses	/	/

Class of recommendation: Class I—evidence and/or general agreement that a given treatment or procedure is beneficial, useful, effective. Level of evidence: A—Data derived from multiple randomized clinical trials or meta-analyses. This table is re-used from authors’ previous work [16].

**Table 2:** ACCP guidelines for the use of aspirin in pregnancy.

Guideline/Comment	Indication	Grade
For pregnant women with prosthetic valves and a high risk of thromboembolism, the addition of low doses of acetylsalicylic acid (75–100 mg daily) has been recommended.	Prophylaxis on mechanical prostheses thrombosis	2C
For pregnant women who meet the diagnostic criteria of antiphospholipid syndrome, the addition of low doses of acetylsalicylic acid (75–100 mg per day) with prophylactic use of heparin has been recommended.	Antiphospholipid syndrome	1B
For pregnant women at risk of preeclampsia, the use of low doses of acetylsalicylic acid has been recommended, starting from the second trimester,	Pre-eclampsia	1B

Grading scheme: Grade 1—strong recommendation; Grade 2—weak recommendation. Level of evidence: Grade B—moderate; Grade C—low. This table is re-used from authors’ previous work [16].

**Table 3:** Summary of the use of antiplatelets in pregnancy and lactation.

Drug	Placenta Crossing	Secretion in Milk	Comment	Use in Pregnancy When Indicated
Aspirin	Yes	Secreted in milk in small quantities, well tolerated	Not enough information for doses higher than 100 mg	Yes, low dose aspirin
Clopidogrel	Unknown	Yes	Only animal studies available	Yes, when no other options
Ticagrelor	Unknown	Yes	Only animal studies available	No, but current case reports show positive results
Prasugrel	Unknown	Yes	Only animal studies available, according to which no malformations were noticed	No, but current case reports show positive results
Tirofiban	Unknown	Unknown	Animal studies have shown some secretion into milk	No, but current case reports show positive results
Eptifibatide	Unknown	Unknown	If therapy is necessary, it is recommended to stop breastfeeding	No, but current case reports show positive results
Abciximab	Unknown	Unknown	Use only if absolute benefit outweighs the risk	Probably no
Cilostazol	Unknown	Unknown	Animal studies have shown reproductive toxicity	No
Dipyridamole	Minimal	Milk concentration approximately 6% of plasma concentration	No clinical studies available but the long experience of the use in pregnant women indicates probable safety	Probably yes

Adapted from Regitz-Zagrosek, V. et al. ESC Guidelines for the management of cardiovascular diseases during pregnancy [1].

## Data Availability

The original contributions presented in the study are included in the article, further inquiries can be directed to the corresponding author.
